# Development of Colonic Polyposis in a Woman With Chronic Myeloid Leukemia Treated With Dasatinib

**DOI:** 10.1155/crh/4605635

**Published:** 2026-04-21

**Authors:** Guido Pelaez, Behram Suha Yildiz, Mert Candan, Mohammad Ismail, Francesca Vacca, Marianna B. Ruzinova, Samuel Ballentine, Kathleen Byrnes, Armin Ghobadi

**Affiliations:** ^1^ Department of Medicine, Washington University School of Medicine in St. Louis, St. Louis, Missouri, USA, wustl.edu; ^2^ Cerrahpasa School of Medicine, Istanbul University-Cerrahpasa, Istanbul, Turkey, istanbul.edu.tr; ^3^ Department of Medicine, Division of Gastroenterology, Washington University School of Medicine in St. Louis, St. Louis, Missouri, USA, wustl.edu; ^4^ Department of Pathology and Immunology, Washington University School of Medicine in St. Louis, St. Louis, Missouri, USA, wustl.edu; ^5^ Department of Medicine, Division of Medical Oncology, Washington University School of Medicine in St. Louis, St. Louis, Missouri, USA, wustl.edu

**Keywords:** case report, colon, dasatinib, leukemia, polyps, tyrosine kinase inhibitor

## Abstract

**Introduction:**

Chronic myeloid leukemia (CML) is a hematopoietic malignancy driven by constitutive tyrosine kinase activity. Dasatinib, a second‐generation (2G) tyrosine kinase inhibitor (TKI), is a preferred treatment due to its superior, durable, and rapid response rates. While dasatinib has known gastrointestinal (GI) side effects, colonic polyposis is a rare complication, not well described in the literature.

**Case Presentation:**

Upon routine colonoscopy, a 66‐year‐old woman with chronic‐phase CML was incidentally discovered to have asymptomatic colonic polyposis after 11 months of dasatinib therapy. Histopathology revealed reactive inflammatory changes without evidence of dysplasia or neoplasia. The polyposis resolved completely after cessation of dasatinib.

**Conclusion:**

The mechanism behind dasatinib‐induced polyposis is unclear. We hypothesize a role of dasatinib’s differential inhibition of multiple kinase pathways, regulatory T cells, and STAT5 signaling in the intestinal epithelium, causing an unregulated inflammatory state. This case underscores the need for awareness of this rare adverse effect and further research into its pathogenesis.

## 1. Introduction

Chronic myeloid leukemia (CML) is a clonal myeloproliferative neoplasm characterized by the t(9; 22) translocation. The resulting Philadelphia chromosome generates the BCR‐ABL1 fusion gene, which encodes for a constitutively activated tyrosine kinase that drives malignant transformation of hematopoietic stem cells [1]. Since the FDA first approved imatinib in 2001, tyrosine kinase inhibitors (TKIs) have long been potent first‐line therapies for CML. Dasatinib is a second‐generation (2G) TKI, which has demonstrated faster, superior, and more durable response compared to imatinib in newly diagnosed chronic‐phase CML [1]. In the DASISION trial, dasatinib exhibited a higher rate of confirmed complete cytogenetic remission (77% vs. 66%) and deeper major molecular responses (46% vs. 28%) at 12 months compared to imatinib [2]. While the drug is associated with lower rates of edema, rash, gastrointestinal (GI), and musculoskeletal adverse effects, it has higher rates of pleural effusion (PE) and thrombocytopenia [3]. Dasatinib‐associated PEs are increasingly understood as immune‐mediated rather than transudative in origin, with pleural fluid analyses typically revealing lymphocyte‐predominant exudates [4, 5]. Adverse GI effects mostly consist of abdominal pain, nausea, and diarrhea; however, more serious and uncommon immune‐mediated presentations, such as hemorrhagic colitis, have been documented [6–8]. Several reports have also described lymphocytosis following dasatinib administration, driven by large granular lymphocyte (LGL) proliferation of cytotoxic (CD8+) T cells and/or natural killer (NK) cells, which has been associated with PE, pleuritis, and colitis [9–11]. However, colonic polyposis attributed to dasatinib has been rarely described in the current literature, and a clear pathological mechanism has not yet been defined [8, 12]. To date, two cases of inflammatory colonic polyposis have been reported, only one of which is published in the English literature [8, 12, 13]. Here, we present the third case to occur in a patient with CML undergoing first‐line therapy with dasatinib. We theorize that LGL lymphocytosis, PEs, colitis, and colonic polyposis may represent different presentations of a possibly singular immune process mediated by dasatinib.

## 2. Case Presentation

A 66‐year‐old Caucasian female with no significant past medical history or family history of malignancy presented asymptomatically to the emergency department at the instruction of her primary care provider due to abnormal routine labs. Her complete blood count (CBC) was notable for significant leukocytosis (270 K/μL, absolute neutrophil count [ANC] = 130 K/μL) with concurrent anemia (Hgb 9.1 g/dL) and thrombocytosis (479 K/μL). Peripheral blood morphology and flow cytometry demonstrated marked neutrophilia with myeloid left‐shift and scattered circulating blasts (< 10%). FISH testing on bone marrow biopsy showed 77.5% atypical BCR/ABL1 rearrangement and 13.5% typical BCR/ABL1 rearrangement. Highly positive BCR/ABL p210 PCR confirmed the diagnosis of chronic‐phase CML. Her initial physical examination was unremarkable, with no significant organomegaly or palpable lymph nodes. Her disease was categorized as intermediate risk based on the Sokal and Euro score. She elected to undergo upfront therapy with oral dasatinib 100 mg daily and quickly achieved hematologic remission within a month of starting therapy. She was routinely followed in the clinic and continued to tolerate the medication well without noticeable major adverse reactions. Eleven months into her treatment, she underwent a routine screening colonoscopy. Multiple 5–10 mm sessile, inflammatory polyps with white exudative surfaces were discovered within the descending and transverse colon (shown in Figure 1). Eight polyps were resected and sent for pathology. The remaining multiple polyps were left undisturbed pending the results. The pathology report disclosed reactive lymphoid follicles and inflammation of the lamina propria with increased neutrophils, eosinophils, and plasma cells (shown in Figure 2). Diagnostic features of lymphoma were notably absent. Her last colonoscopy 5 years prior was normal. The patient was counseled on the risks and benefits of switching vs. continuing dasatinib and ultimately arrived at the decision of continuing the medication and repeating the colonoscopy in 6 months. Her repeat colonoscopy again revealed multiple inflammatory‐appearing, subcentimeter sessile polyps in the sigmoid, descending and transverse colon (shown in Figure 1). Multiple polyps were removed from the sigmoid and descending colon. Five polyps were removed from the transverse colon. As per the procedure note, due to the number of polyps present, the majority were not resected. Pathology redemonstrated identical reactive inflammatory features without evidence of lymphoma (shown in Figure 2). Given her persistent polyposis, she was transitioned to oral nilotinib 300 mg twice daily after 17 months of therapy with dasatinib. Repeat colonoscopy 11 months after switching dasatinib to nilotinib showed mildly erythematous, nonulcerated mucosa limited to the cecum but was otherwise normal and devoid of polyps (shown in Figure 1). Biopsies were taken for pathology, which revealed normal colonic mucosa. Her most recent clinic follow‐up was 13 months since transitioning to nilotinib, during which she reported feeling generally well with only mild constipation that was well managed with stool softeners. Her CBC continued to demonstrate hematologic remission, and she achieved molecular remission MR4.5 (positive BCR/ABL PCR with BCR/ABL to ABL1 ratio < 0.0032%).

FIGURE 1Colonoscopy images demonstrating presence and resolution of inflammatory polyps in the distal colon. (a) Approximately 10 months of dasatinib therapy. There are multiple sessile polyps with white exudative heads. (b) Repeat colonoscopy after 6 months with persistent inflammatory sessile polyps. (c) Repeat colonoscopy after 18 months demonstrating complete resolution of polyps with return to normal‐appearing mucosa.

**Figure:**
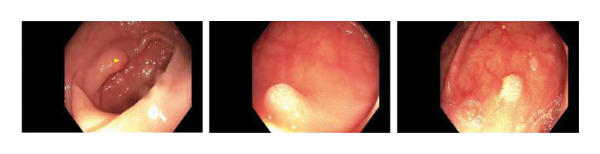
(a)

**Figure:**
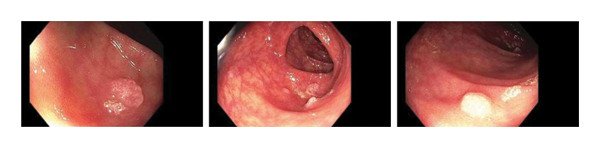
(b)

**Figure:**
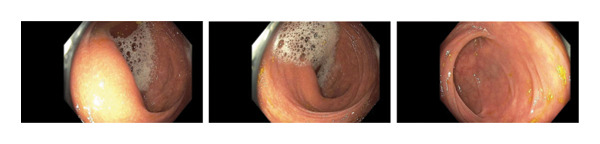
(c)

FIGURE 2(a) Initial colonoscopy tissue biopsy. Hematoxylin and eosin, 100 ×. Colonic mucosa demonstrates a brisk mixed inflammatory infiltrate that expands the lamina propria. Rare intraepithelial lymphocytes and neutrophils are present in glands that overlie a lymphoid follicle with a germinal center. (b) Immunohistochemistry and in‐situ hybridization were performed to further characterize the inflammatory infiltrate, 100 ×. CD3 highlights small mature T‐cells, primarily on the periphery of the lymphoid follicle, while CD20 highlights B‐cells, primarily in the follicle’s center. BCL2 is negative within the germinal center. BCL6 and CD10 appropriately highlight B‐cells within the germinal center and are largely negative at the periphery. CD21 appropriately highlights the follicular dendritic cell meshwork. Kappa and lambda in‐situ hybridization demonstrate a polytypic population with an appropriate ratio between kappa and lambda. Ultimately, these markers demonstrate normal germinal center architecture, appropriate for a reactive lymphoid follicle. (c) Repeat 6‐month colonoscopy tissue biopsy. Hematoxylin and eosin, 40 × and 400 ×. Colonic mucosa demonstrates polypoid architecture, characterized by a brisk inflammatory infiltrate that expands the lamina propria. A lymphoid follicle is present toward the base. The inflammatory infiltrate consists of a mixed inflammatory population including lymphocytes, plasma cells, histiocytes, eosinophils, and neutrophils. A few inflammatory cells are present within the glandular epithelium. (d) Colonoscopy tissue biopsy 11 months after switching to nilotinib. Hematoxylin and eosin, 40 × and 200 ×. Colonic mucosa demonstrates flat architecture without increased inflammation.

**Figure:**
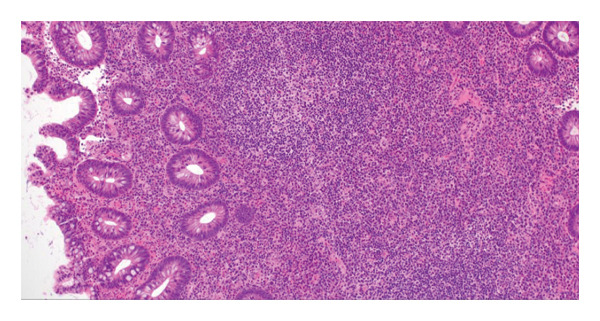
(a)

**Figure:**
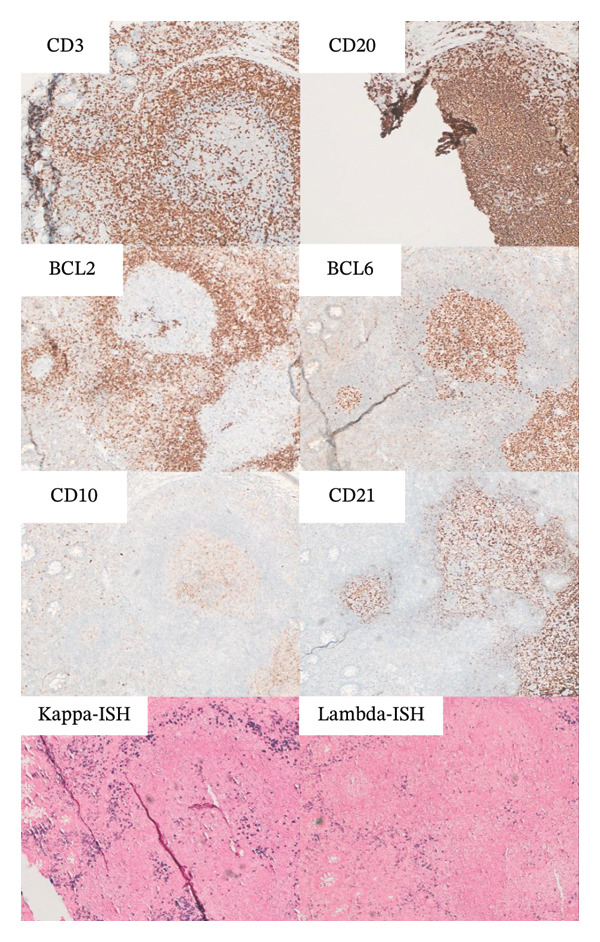
(b)

**Figure:**
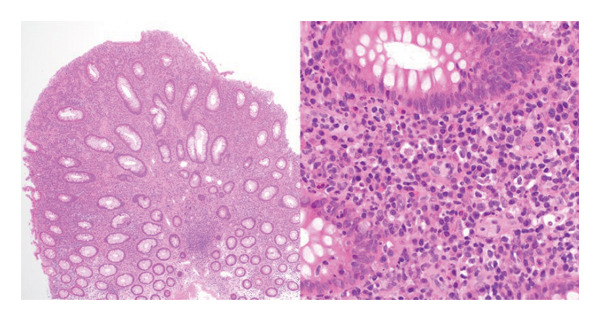
(c)

**Figure:**
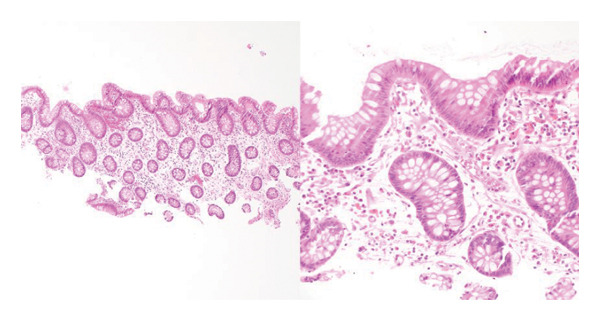
(d)

## 3. Discussion

Treatment of CML largely centers on achieving durable clinical, cytogenetic, and molecular remission, as well as preventing progression to advanced disease [1]. The National Comprehensive Cancer Network (NCCN) guidelines for CML report no significant differences in overall survival in patients who start imatinib versus a 2G TKI [1]. The guidelines recommend both generations for first‐line therapy in patients with new low‐risk CP‐CML [1]. However, in intermediate or high‐risk CML, 2G TKIs are the preferred agents given that they are associated with a lower risk of disease progression and faster molecular responses as compared to imatinib [1].

In the case of our patient, dasatinib was the preferred first‐line therapy given its once daily dosing and our patient’s intermediate‐risk disease. Ultimately, it was well‐tolerated without major side effects apart from the numerous inflammatory polyps present in her descending and transverse colon that were incidentally found during routine colonoscopy. Given the number of polyps present, this was consistent with a diagnosis of colonic polyposis, which persisted while on dasatinib but cleared completely after transitioning to nilotinib. Interestingly, the histology of the biopsied polyps more closely resembles a reactive inflammatory state (as evidenced by reactive lymphoid follicles, some with germinal centers) rather than a neoplastic/dysplastic process. It can be surmised that dasatinib may act as a proinflammatory mediator at the level of the colonic mucosa in ways that other TKIs do not. While our patient demonstrated a return to normal on repeat histology 12 months after stopping dasatinib, it remains unclear whether chronic exposure to dasatinib has the potential to mediate the emergence of lymphoid neoplasia, as would be the case in other MALT lymphomas.

The phenomenon of inflammatory colonic polyposis may represent a manifestation of a broader spectrum of immune‐mediated complications induced by dasatinib. Beyond inhibiting BCR‐ABL1 kinase activity, dasatinib is a potent inhibitor of other oncogenic kinases. These include c‐KIT, platelet‐derived growth factor receptor (PDGFR), and Src family kinases (SFKs) [6], which are critical regulators of immune response. Dasatinib therapy has been demonstrated to significantly deplete regulatory T cells (T‐regs) and encourage clonal proliferation of autoreactive effector cells, specifically CD8+ T cells and NK cells [6, 10, 11, 14]. Several reports describe a proportion of patients on dasatinib therapy who develop lymphocytosis with LGL morphology [15]. Interestingly, LGL lymphocytosis is strongly associated with the development of PE, which is typically exudative with a lymphocytic predominance [2, 4, 5, 16]. Immunophenotyping and molecular genetic analysis of these exudates demonstrate similar phenotypes and genotypes present in co‐occurring or preceding lymphocytosis [14]. Dasatinib‐related follicular hyperplasia has also been described, manifesting as palpable lymphadenopathy with microscopic findings of enlarged germinal centers that are immunoreactive to CD3 and CD10/CD20, but not BCL2 [17, 18]. These reports resemble similar histology to that found in our patient and similarly resolve following dasatinib discontinuation [17, 18].

In addition, there may be an exposure‐dependent disruption of intestinal epithelial or immune cell signaling pathways via nonspecific inhibition of multiple proteins with tyrosine kinase domains that regulate intestinal homeostasis. Comprehensive reviews on TKI side effects revealed that TKI‐associated diarrhea is most likely caused by blocking intestinal epidermal growth factor receptor (EGFR) and limiting mucosal healing [19–21]. The BRAF and MEK pathways may also be implicated. TKIs with greater blocking properties of these pathways (mostly 2G irreversible inhibitors) have been associated with higher GI side effects [21]. Nonetheless, these properties are not entirely specific to dasatinib and therefore may not fully explain the polyposis associated with the drug.

We further suspect that dasatinib’s differential inhibition of T‐regs and STAT5 signaling at the level of the intestinal epithelium is a contributing mechanism. Dasatinib has been shown to selectively inhibit T‐cell receptor and STAT5 signaling pathways, leading to depletion of effector T‐regs and an increase in effector CD8‐positive T‐cells [22]. We hypothesize that this may lead to an unregulated inflammatory state that is further driven by dysregulation of intestinal epithelial stem cells (IESCs). IESC proliferation is critically modulated by the STAT5 pathway and has been demonstrated to mitigate intestinal inflammation [23].

Whatever the mechanism is that leads to the formation of these inflammatory polyps, it is important to educate patients on this rare adverse effect of dasatinib. Here, we describe only the third known case; however, it is highly likely that this complication is going unknown in many patients being treated with CML. More research should be performed in discerning the pathogenesis of these colonic polyps and revealing any unifying mechanism that may result in other immune‐mediated adverse effects associated with dasatinib.

## Author Contributions

Armin Ghobadi and Mohammad Ismail treated the patient. Francesca Vacca, Marianna B. Ruzinova, Samuel Ballentine, and Kathleen Byrnes prepared and reviewed the colonic biopsy histology slides and provided the final pathology reports. Armin Ghobadi, Guido Pelaez, Mert Candan, and Behram Suha Yildiz participated in the conception of the manuscript. Guido Pelaez, Mert Candan, and Behram Suha Yildiz interpreted the clinical information and results and contributed to the literature review. Armin Ghobadi had full access to all of the data in this study and takes complete responsibility for the integrity of the data and the accuracy of the data analysis.

## Funding

This case report was not supported by any sponsor or funder.

## Disclosure

All authors have read and approved the final version of the manuscript.

## Ethics Statement

This case report has been submitted for review for human subjects research determination by the Washington University in St. Louis Institutional Review Board. It has been determined to not meet the definition of human subjects research as described in 45 CFR 46.102(I). Written informed consent was obtained from the patient for publication of the details of their medical case and any accompanying images.

## Consent

Please see the Ethics Statement.

## Conflicts of Interest

The authors declare no conflicts of interest.

## Data Availability

All data analyzed for this case report were obtained from patient’s electronic medical record and are included in this article. Further inquiries can be directed to the corresponding author.
